# Development and assessment of the feasibility of a Zika family support programme: a study protocol

**DOI:** 10.12688/wellcomeopenres.15085.1

**Published:** 2019-05-13

**Authors:** Antony Duttine, Tracey Smythe, Miriam Ribiero Calheiro de Sá, Silvia Ferrite, Maria Elisabeth Moreira, Hannah Kuper

**Affiliations:** 1 London School of Hygiene & Tropical Medicine, London, UK; 2 Instituto Fernandes Figueira (IFF), Rio de Janeiro, Brazil; 3 Department of Hearing and Speech Sciences, Federal University of Bahia, Salvador, Brazil

**Keywords:** Zika, disability, microcephaly, early intervention, congenital Zika syndrome, family, caregiver, Brazil

## Abstract

The Zika virus outbreak in Brazil in 2015 affected thousands of people. Zika is now known to cause congenital malformations leading to impairments and developmental delays in affected children, including Congenital Zika Syndrome (CZS). Children with CZS have complex care needs. Caregivers require significant levels of support to meet these needs, and there are large gaps in healthcare services.

This study aims to develop, pilot and assess the feasibility and scalability of a community-based Family Support Programme for caregivers of children with CZS. The programme is adapted from the Getting to Know Cerebral Palsy (GTKCP) programme for the context of CZS in Brazil. GTKCP is a 10-session programme held with 6-10 caregivers in the local community. It includes practical, educational, peer-support and psychosocial aspects, which aim to improve confidence and capacity to care for a child with CP, and quality of life and empowerment of caregivers.

The research project contains four components:

1. Ascertaining need for the caregiver programme: a mixed-methods approach that included two literature reviews, interviews with key stakeholders in country, and incorporation of findings from the Social and Economic Impact of Zika study.
2. Adapting GTKCP for the context of CZS and Brazil: undertaken with guidance from technical experts.
3. Pilot testing the intervention: deliver the 10-session programme to one group of caregivers of children with CZS in Rio de Janeiro and another in Greater Salvador.
4. Update the manual through fast-track learning from participant and facilitator feedback. Assessing the feasibility of the intervention for scale up: deliver the updated programme to two groups each in Rio de Janeiro and Greater Salvador, and evaluate the acceptability, demand, implementation, practicality, adaptation, integration, expansion, and limited efficacy, through questionnaires, direct observation, semi-structured interviews and cost calculation. The project has ethics approval in both the UK and Brazil.

## Introduction

Although several outbreaks of the Zika virus have occurred across the world since it was first identified in the 1950s, it was not until the sudden increase in numbers of cases were recorded in Brazil in 2015 that Zika started to garner significant international attention ^1^. Some 6 months after the first Brazilian cases, a spike in cases of microcephaly was noted and caused Brazil and the international health community to question whether there was a link between Zika and birth anomalies ^2^. Prior to that outbreak, Zika was thought to be relatively innocuous, causing few hospitalisations and was not believed to be fatal ^3^. Zika was declared a Public Health Emergency of International Concern by the WHO in February 2016. This declaration was lifted in November 2016 with the recognition that Zika is likely to remain an ongoing challenge for the public health community and that *“a robust longer-term technical mechanism was now required to manage the global response”* ^4^. The link between Zika and congenital conditions has now been proven ^5^ and the group of impairments and developmental delays in affected infants and young children is known as “Congenital Zika Syndrome” (CZS) ^6^.

CZS is a recognizable pattern of structural anomalies and functional impairments secondary to central and perhaps peripheral nervous system damage. In describing CZS, Moore *et al.* ^6^ suggested five unique features:

- Severe microcephaly with partially collapsed skull;
- Thin cerebral cortices with subcortical calcifications;
- Macular scarring and focal pigmentary retinal mottling;
- Congenital contractures; and
- Marked early hypertonia and symptoms of extrapyramidal involvement.

More recent evidence suggests that not all children with developmental issues relating to Zika have CZS or present with microcephaly at birth. Some are born with a normal head circumference and go on to develop microcephaly later, and others show evidence of the other features without microcephaly ^7^. Microcephaly, therefore may be the tip of the iceberg with regards the wider array of clinical and developmental features ^8^. Consequently, the approximately 3000 cases of microcephaly with confirmed Zika infection may dramatically underestimate the true scale of the condition in Brazil. Additionally, it is not known what health conditions or impairments may yet manifest in young children as they continue their development. The oldest group of children from the Brazilian outbreak are 3 years old as of January 2019. For the purposes of this paper and project, we used CZS to describe any child with impairments that can be directly attributed to Zika.

What is known is that children with CZS are likely to require ongoing support and care from the health, social, education and other sectors as they grow and develop ^9– 13^. Families experience heavy burdens of care raising children with similar neuro-developmental disabilities, such as cerebral palsy (CP) ^14^. A few studies have already shown high levels of anxiety amongst mothers of children with CZS ^15, 16^.

In spite of the disabling impact of Zika, only a fraction of funding and research has been focussed towards meeting the care and support needs of children with CZS and their families. Perhaps reasonably, most programmes have targeted comprehending the nature of the virus in order to work towards a vaccine and future prevention ^17^. Meanwhile, the health services for children with neuro-developmental disabilities that exist in Brazil, including physiotherapy, occupational therapy, speech therapy and other therapeutic services, alongside medical services, have been overwhelmed by an upsurge in demand ^18, 19^. Against a background of generally stretched services, there is also inequity in the availability of these services, particularly in non-urban areas ^20^, and the non-clinical needs are often overlooked. Families often have only sporadic, limited or unstructured education and support with respect to the care of their child. True health promotion, however, requires meeting the holistic needs of families ^21^.

**Figure 1.**
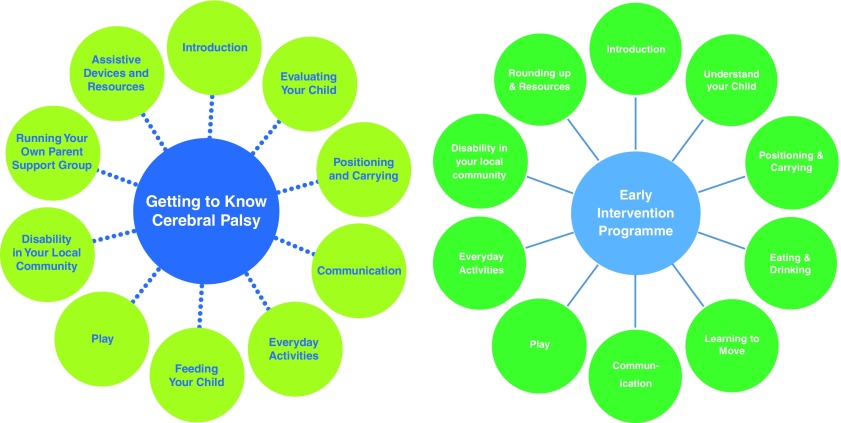
Structure of Getting to Know Cerebral Palsy and the Early Intervention Programme.

A similar situation of unmet healthcare needs and unsupported families is also apparent for other types of complex childhood disability, such as CP. In response to this recognised need to provide for the holistic care of families of children with complex multiple impairments the London School of Hygiene & Tropical Medicine developed a participatory caregiver group programme for children with CP called ‘Getting to Know Cerebral Palsy’(GTKCP) ^22^ ( Figure 1). The programme sought to educate and empower these caregivers to optimise their situation, quality of life and the ability for their child to maximise his/her potential to participate within society. It was developed and pilot-tested in Bangladesh.

*GTKCP* has since been implemented in over 25 countries. In Ghana, an evaluation showed that the programme was positively received by families and had a positive impact on both quality of life and knowledge and confidence of caregivers of children with CP, as well as the reported physical and emotional health of the child ^23^. The programme was originally designed for children of age 2 and over, and a recent adaptation has been piloted in Uganda for children under 2, the Early Intervention Programme (EIP), and is currently being clinically trialled ^24^.

This current study aims to develop, pilot and assess the feasibility and scalability of a community-based *Family Support Programme* for caregivers of children with CZS. The programme is adapted from GTKCP for the context of children with CZS in Brazil. The specific objectives of the research are:

1. To undertake a needs assessment for the intervention
2. To adapt GTKCP and EIP for the context of CZS in Brazil.
3. To conduct a pilot programme in two sites in Brazil for families of children with CZS.
4. To assess the feasibility of the pilot programme for potential scale up and roll out across the country and beyond.

## Protocol

### Overview of study design and setting

The Family Support Programme is the implementation arm of two research initiatives undertaken by the International Centre for Evidence in Disability at the London School of Hygiene & Tropical Medicine. The sister study is a mixed methods evaluation of the social and economic impacts of CZS that took place concurrently ^25^ and data fed into the content of the Family Support Programme.

Two locations in Brazil were used to pilot the programme - Rio de Janeiro and Salvador, both of which had high numbers of Zika cases and children born with CZS ^9^. The study is undertaken in partnership with Fundação Oswaldo Cruz (known as Fiocruz), the national institute for health research. In Rio de Janeiro, the partner was the National Institute of Women, Children and Adolescent Health Fernandes Figueira (IFF), part of Fiocruz, and in Salvador it was the Federal University of Bahia.

Ethics approval was acquired in both Brazil (IFF/FIOCRUZ - RJ/MS 2.183.547) and the UK (LSHTM Ethics number 13608). All participants who took part in the programme completed a consent form ^26^, relevant to their involvement in the study (e.g. survey, interview). Participants were also requested to provide consent for photographs or other media to be recorded during the group sessions, once it was explained that non-agreement to the media consent form would not impact their position in the groups.

Below we describe the methods for the four objectives:

***1. To undertake a needs assessment for the intervention***

A mixed-methods approach was adopted to identify the needs for a family support intervention. This approach included reviewing emerging and associated literature, an in-country needs assessment with qualitative investigations, and incorporation of findings from the sister study ^25^.

A scoping review was undertaken in May 2017 to describe the clinical presentation of Zika-related impairments in children, including CZS and its similarities and differences with other neurodevelopmental disabilities. Studies published between October 2015 and April 2017 (i.e. since the onset of the Brazil outbreak) on CZS were identified through PubMed searchers using ‘Zika’, ‘Microcephaly’, ‘Congenital Zika Syndrome’ as search terms and reviewing the reference list of relevant papers. Data and evidence that contributed to information about the clinical presentation of CZS was compiled and presented to inform the programme structure, though the paper by Moore *et al.* ^6^ provided a timely and comprehensive overview of the research and clinical features of CZS. These summaries were then compared to literature on the clinical presentation of other neurodevelopmental disabilities, most notably CP, guided by a paediatric neurologist.

A literature review was undertaken in summer 2017 to explore the implications for CZS and CP for support needed for affected families. Search terms included 'zika virus', 'congenital zika syndrome', ‘cerebral palsy’, ‘family needs’, ‘parent needs’, ‘psychosocial’, ‘cost’, economic impact’ alone and in combination were used in CINAHL Plus, EMBASE, MEDLINE, PsychInfo, and PubMed with formats for search terms adapted for different databases. Eligibility criteria included any study published in peer reviewed journals that described the needs of families of children affected by CZS and/or microcephaly related to Zika, or CP (in low-middle-income countries only) and was published in English language in peer-reviewed journals between January 2000 and July 2017.

A scoping needs assessment was undertaken by members of the researcher team in Brazil in April 2017. The scoping visit involved reviewing the current context in Brazil and the project sites, in terms of structure, function and availability of health and social services that may be needed by parents, to ascertain the needs of the intervention. This assessment was undertaken by meeting with a range of clinicians in both Rio de Janeiro, Salvador and Recife, and included doctors, therapists, psychologists. Furthermore, meetings with organisations working to support families with children with CZS and informal consultations with families themselves were also undertaken. Meetings and discussions were not recorded, but annotated.

Finally, findings from the Social and Economic Impact of Zika study ^25^, funded by the Wellcome Trust, were incorporated into the needs analysis. This included data from semi-structured interviews ^27^ with families of children with CZS to ascertain their needs and the impact of CZS on their lives, specifically assessing need for psychological, social, financial and other supports. Data were provided through direct dialogue with the team and from published papers.

The team members considered evidence across these four sources to identify where the needs and service gaps were most substantial, which would need to be targeted by the intervention.

***2. To adapt the GTKCP and EIP for the context of CZS in Brazil***

The Family Support Programme is based on the existing structure of GTKCP ^23^ and the EIP ^24^, as no other relevant interventions for this target group were identified. Findings from the needs assessment, described above, were used to adapt the programme to meet the specified and identified needs of caregivers of children with CZS in Brazil and gaps in services (May–July, 2017). These adaptations were undertaken by a project team, which included individuals in Brazil and globally, with expertise in care for children with complex needs and their families, such physiotherapists, paediatricians and social scientists. The team also included individuals who developed the GTKCP and EIP. One team member was assigned the role to lead on content development (AD). External support was sought where needs were identified that were outside of the expertise of the group (e.g. nutrition).

Further, two technical advisory groups (TAG) were established—one in Brazil and one in the UK. These groups contained members with a diverse background and experience related to Zika including researchers, health professionals and parent advocates. The role of the TAGs was to provide input and feedback during the development, review and finalisation of the programme. Most notably, the required tasks were to agree on the structure of the initial pilot programme, to review and agree on the changes made between phase 1 and 2 (see below) and review and agree on the final proposed programme after all pilot groups are concluded. The TAGs were also consulted on sample size and inclusion criteria, facilitator and researcher profiles, module structure, session frequency and other programme parameters.

The main implementation element of the programme is to deliver a series of sessions to a group of caregivers. Materials were produced to support the programme including a manual for the facilitators providing guidance for the Family Support Programme content and structure, and images printed on durable textile for group work.

***3. To conduct a pilot programme in two sites in Brazil on families of children with CZS***

Once the initial adaptation of the programme was agreed by the two TAGs, it was pilot-tested within two groups in Brazil (August–November, 2017).

Eight facilitators for the caregiver groups were identified by the site coordinators (M.S. and S.F.), and included four mothers of children with CZS and four therapists (e.g. speech and language). Facilitators were enrolled in a training programme in July 2017, to be educated and capacitated on the content of the programme and aspects on delivering participatory groups. An expert consultant was used for this process, who had experience from the GTKCP programmes. The training programme lasted 5 days and included orientation to the content of the course, facilitation on adult based learning styles and practice sessions.

Subsequently, one parent group was established in Rio de Janeiro, and one in Greater Salvador. The sessions were guided by the facilitators through the structure and order of the adapted programme. Caregivers were identified using criteria agreed during the UK TAG—families of children with confirmed or suspected CZS who were residing at home (i.e. not receiving inpatient hospital care at the time of the start of the programme), who agreed to be involved in the programme and who were not participating in a conflicting group study (but could be receiving individual therapy). Participants were identified through clinical and therapy networks at the two sites and identified participants were contacted by site coordinators about joining the groups. Each group included 6–10 caregivers of children with CZS, and was held approximately weekly in the local community, for 10 sessions. Each session lasted approximately 3 hours, and a range of topics were covered (e.g. feeding, play, communication).

Researchers received pre-pilot training to review and familiarise with the questionnaires, observation and focus group procedures and interview schedules. Two researchers were assigned to each group and held focus group discussions with participants and facilitators at the end of each session to record data on a pre-designed format.

Fast-track learning was used during the pilot testing to hone and adjust the structure and content of the programme from the initial draft. Two main sources of information were utilised for Fast track learning:

- Researcher observation: researchers attended the sessions to observe and acquire feedback from participants and facilitators. Researchers observed the sessions using a checklist guide without directly intervening in the group. The completed form was sent to the coordinators (A.D. and T.S.) after each session, in English. The checklist contained information about the participatory approaches used by facilitators, the level of interest and engagement of participants, and noted any aspects that went well or did not go well and require modification or improvement.
- End-of-session focus groups

The programme content and delivery was updated based upon the feedback received.

Facilitators and researchers received compensation for their involvement in the programme. The amount of compensation was ascertained and managed by the Fiocruz partners in Brazil based on their allocated budget. Participants did not receive any financial compensation for taking part in the programme.

***4. To assess the feasibility of the pilot programme for potential scale up and roll out across the country and beyond***

A further 3-day ‘updating’ training was undertaken in December 2017 to provide facilitators with information on the changes to the content and structure of the programme based on fast-track learning in the pilot phase. Two further parent groups were established in each setting as above, to ascertain the feasibility of the intervention, with identical procedures for fast-track learning and data collection (February–June, 2017).

The feasibility of the programme was assessed through the eight areas of focus proposed by Bowen *et al*. ^28^ for evaluating public health interventions ( Table 1).

**Table 1.** The eight areas of focus. Adapted from Bowen *et al* ^28^.

Area of focus	The feasibility study asks…
Acceptability	***“*** *To what extent is a new idea, program, process or measure judged as suitable, satisfying, or attractive to program* *deliverers? To program recipients?”*
Demand	***“*** *To what extent is a new idea, program, process, or measure likely to be used (i.e., how much demand is likely to* *exist?)”*
Implementation	*“To what extent can a new idea, program, process, or measure be successfully delivered to intended participants in* *some defined, but not fully controlled, context?”*
Practicality	***“*** *To what extent can an idea, program, process, or measure be carried out with intended participants using existing* *means, resources, and circumstances and without outside intervention?”*
Adaptation	***“*** *To what extent does an existing idea, program, process, or measure perform when changes are made for a new format* *or with a different population?”*
Integration	***“*** *To what extent can a new idea, program, process, or measure be integrated within an existing system?”*
Expansion	*To what extent can a previously tested program, process, approach, or system be expanded to provide a new program* *or service?*
Limited efficacy	**“** *Does the new idea, program, process, or measure show promise of being successful with the intended population,* *even in a highly controlled setting?”*

In total, four sources of data were collected to give an overall valuation of the programme and provide the appropriate information for the assessment into the feasibility. These include collecting data from participants of the programme, facilitators and other key stakeholders:

### Participant data

Pre- and post-programme semi-structured questionnaires ^29^ were completed by all participants in the programme before the first session and after the final session of each group. This data was then logged into a password-protected Google Drive® document, shared with the content development lead, and discussed between the researcher and content lead within two days of submission. Questionnaires were developed in English and translated into Portuguese, and included the following items:

- Socio-demographic characteristics of the child and caregiver ^1^.
- Perceived unmet needs and main goals for the intervention ^1^.
- The PedsQL™ Family Impact Questionnaire Module ^30^ (using the official version translated to Brazilian Portuguese).
- Understanding and knowledge about the child’s condition.
- Self-reported functioning of the child.
- Nutrition and feeding and drinking practices
- Subjective well-being of caregiver and child
- Review of goals achieved ^2^
- Satisfaction with programme ^2^

Minor adjustments to the translation and structure of the questionnaires was made by the researchers after the pilot groups to improve certain sections. Questionnaires were pilot-tested in a sample of participants. Researchers administered the questionnaire to participants before commencing the first group and after completing the final group. Participants should have attended a majority of sessions (>50%) to complete the final questionnaire. Attendance of sessions was monitored through a simple registry.

The PedsQL™ Family Impact Questionnaire Module ^30^ was selected for a number of reasons. First, it contains a range of measures where we anticipated impact by the programme, for example emotional functioning, worry. Second, it has been validated in Brazilian Portuguese ^31^. Third, it is also being applied in the sister study ^25^, which allowed some consistency and comparability for further analysis.

Semi-structured interviews were undertaken with two or three participants per group within 15 days of the final session of each group either in the setting of the group meetings or at the participant’s home. Participants were selected at the discretion of researchers to reflect a broad a range of perspectives (e.g. caregivers of children with different severities of disability, mothers and fathers). Interviews were undertaken in Portuguese by the local researchers, asking about satisfaction with and perceived impact of the groups. The interviewer recorded and transcribed the interviews.

### Facilitator data

A semi structured interview of up to 30 minutes was undertaken at the location of the group sessions with each of the facilitators (total, seven) at the final session of the final group reflecting the whole process. These were undertaken by the local researchers, who recorded and transcribed the interviews.

### Key stakeholder data

Semi-structured interviews were also conducted with identified key stakeholders in Brazil in April 2018. These included the two site coordinators and involved specific questions around practicality, adaptation, integration and expansion Bowen’s areas of feasibility. Interviews were undertaken in English or Portuguese by either the study leads (AD/TS) or the local researchers, and were transcribed.

### Other data

Cost of the sessions were assessed by analysing the budget and establishing an overall cost for delivery of the programme, and in addition the cost per participant. Training of facilitator costs were calculated and presented separately as this may not be reflective of the structure of a true training of facilitator programme if scaled up (number of facilitators, international travel etc). A costed plan for scale up was considered.

## Data management and analysis

Data was sent to the research team in London by the site coordinators. Interviews were saved as word files and questionnaires in excel. All stored data was anonymized and password protected.

Data was stored by project site (Rio and Salvador) and by group number (i.e. Rio 1, Rio 2, Rio 3, Salvador 1, Salvador 2, Salvador 3). Each participant was given a unique number for pre-questionnaires, session notes and post-questionnaires. Participants interviews were not linked to their individual questionnaire responses.

Analysis of the interviews and session notes/focus groups was undertaken using NVIVO 12® software. A social scientist fluent in English and Portuguese coded the interview responses in NVIVO 12. Thematic analysis was structured around the eight areas of feasibility described by Bowen *et al*. ( Table 1) ^28^ with an additional ‘other’ theme for information that the analyst found pertinent but did not fit into the eight feasibility themes. Analysis of the questionnaires was undertaken using Microsoft Excel, producing data on demographics of participants, change between baseline and endline in the areas described and reflection on the program.

As per Wellcome Trust data management plans, the data collected from this study will be made openly available to specific users (i.e. researchers in an academic environment) on request to the study lead (Antony Duttine) through e-mail ( antony.duttine@lshtm.ac.uk). Data can be analysed only for the specific purposes compatible with the consent agreement. The data is not freely and open available since the sample size is relatively small and even though data is anonymised, there is a risk of establishing the identities of participants.

## Dissemination of findings

A minimum of three additional papers are anticipated from the completion of the research: one on the needs of such an intervention, one on the feasibility analysis and one describing the whole intervention and final programme. Additional areas of potential interest which may be explored are the findings on using a mother as a facilitator and the engagement of fathers in the programme.

The subject is of interest to both the general public and the public health community given the attention that Zika gained. Therefore, it may be likely that there are opportunities for developing grey literature e.g. blog articles, media pieces regarding the work.

Opportunities for submitting abstracts and presenting the work at national (UK and Brazil) and international forums will be pursued. Dissemination events will be arranged in UK and Brazil, inviting key stakeholders.

## Data availability

### Underlying data

All data underlying the results are available as part of the article and no additional source data are required.

### Extended data

Researchgate: Pre and post questionnaires. https://doi.org/10.13140/RG.2.2.13700.17287 ^29^.

Researchgate: Qualitative interview questions for participants, facilitators and key informants. https://doi.org/10.13140/RG.2.2.28380.23686 ^27^.

Researchgate: Consent forms (interviews and questionnaires). https://doi.org/10.13140/RG.2.2.30057.95845 ^26^.

Extended data are available under the terms of the Creative Commons Attribution 4.0 International license (CC-BY 4.0), excluding the PEDS QL instrument which is © 1998 JW Varni, Ph.D. All rights reserved.

## Notes

^1^Only asked at baseline

^2^Only asked at end-line
