# Supplementary material for: Development and assessment of the feasibility of a Zika family support programme: a study protocol
Source: Wellcome Open Res. 2019 May 13;4:80. [Version 1] doi: 10.12688/wellcomeopenres.15085.1 (PMC6600857; doi:10.12688/wellcomeopenres.15085.1)
Supplement: Peer review history

# Peer review: Reviewer response for version 1

**DOI:** 10.21956/wellcomeopenres.16458.r35511

**Published:** 2019-06-28

**Authors:** Michael J. Griffiths

**Affiliations:** 1 Institute of Infection and Global Health, University of Liverpool, Liverpool, UK; 2 Department of Neurology, Alder-Hey Childrens NHS Trust, Liverpool, UK; 3 NIHR Health Protection Research Unit in Emerging and Zoonotic Infections, University of Liverpool, Liverpool, UK

This manuscript entitled  “Development and assessment of the feasibility of a Zika family support programme: a study protocol” outlines a protocol to:

1. undertake a needs assessment on whether Brazilian caregivers of children with congenital zika syndrome may benefit from a family support programme
2. adapt two existing family support programmes , Getting to Know Cerebral Palsy (GTKCP) and Early Intervention Programme (EIP), for use among the above families
3. pilot these adapted programmes among two geographical distinct groups of caregivers in Brazil  and alongside conduct an evaluation of the programme
4. re-pilot the refined adapted programme and alongside undertake a more detailed evaluation of the programme, including an assessment of the feasibility of the programme for potential scale up and roll out across the country and beyond.

I enthusiastically support the premise of the study. Such programmes need to be developed and rolled out among such patient groups and in such settings. On the whole the study protocol is well described and sufficiently detailed.

 I offer the following comments to the author team:

 Echoing the reviewer Michel Landry, the title could be changed to better describe the study as follows ‘Adaptation and assessment of a family support programme for use among caregivers of children with congenital zika syndrome: a study protocol”. This change would highlight that an existing programme was adapted to the target population rather than developed from scratch.

 In the introduction, the initial description on the unique features of congenital Zika syndrome (CZS) as described by Moore and colleagues is probably not really necessary. The study does not recruit their target population based on these features. As the authors state, it has been reported that neurodevelopmental problems can occur in children of mothers exposed to Zika virus during pregnancy that do not exhibit these features.

 In the description of objective 2, the authors appear to ’jump’ to selecting the existing GTKCP and EIP programmes. It would have been useful to describe in more detail why GTKCP and EIP programs were selected and why the authors believed these programmes were appropriate for use in the target group. For example the authors could have mapped the domains of need covered in these programs against the needs of caregivers of CZS children. This would have provided evidence to support the selection.

 In the introduction, the authors could have described the range of neuro-developmental difficulties CZS children have been reported to experience in Brazil and how these difficulties overlap with other neurodevelopmental disorders , such as cerebral palsy. This comparison would have helped to orientate the readers to understand why the authors went on to choose to adapt a family support programme for caregivers of children with cerebral palsy.

 As part of the description of objective two, it would be useful to have access to examples of the adapted GTKCP and EIP programmes in the extended data sets. As a minimum, a few examples of how the programmes’ content or structure were adapted would be helpful.

 In the description of objective four, ‘ *To assess the feasibility of the pilot programme for potential scale up and roll out across the country’ *the authors list a broad series of measures evaluated (including PedsQL, Nutrition and feeding and drinking practices). Several of these measures do not appear to relate to programme feasibility or scale-up. This objective could be written more clearly to explicitly state what measures and how the stated objective was assessed.  

 In the description of objective four, it would be useful to know how the researchers ensured the participants felt free to express any potential negative views of the course (or facilitators), particularly as some interviews were conducted at the programme site?

 Details of the compensation given to facilitators would also be useful. Again this raises the question, how did the researchers/study design ensure compensation (e.g. if compensation was higher or lower than expected) didn’t influence the facilitators’ view of the programme.

 At the start of the methods, the authors state 8 facilitators were trained. In objective four, they state 7 facilitators were interviewed. Details of why one facilitator was not interviewed would add completeness. 

 Echoing the reviewer Michel Landry, I fully support the focus on protection of personal identifiable information. However, I would also urge the authors to reconsider providing open access to the high level aggregated data.This would help provide context to the work on this protocol. 

 Overall this is a very useful study protocol. I very much look forward to the downstream publications.

I confirm that I have read this submission and believe that I have an appropriate level of expertise to confirm that it is of an acceptable scientific standard.

# Peer review: Reviewer response for version 1

**DOI:** 10.21956/wellcomeopenres.16458.r35510

**Published:** 2019-05-20

**Authors:** Michel D. Landry

**Affiliations:** 1 Duke Global Health Institute (DGHI), Duke University, Durham, NC, USA

This manuscript very nicely outlines a protocol that was implemented to explore the effects of Congenital Zika Syndrome (CZS) in Brazil, and the author team was inclusive of colleagues from 1 UK based institution and 2 independent institutes from Brazil.  Ultimately the research team sought to describe their mixed-method approach to address the following areas/questions: is there a need for a caregiver program, and can a previously developed program (GTKCP) be adapted in Brazil for this population. Further, they sought to pilot test an adapted GTKCP, and update a manual (policies, procedures, structed and other such details I expect) related to the adapted program. The description of the protocol was detailed, and I offer the following suggestions to the author team.

1. It would seem to me that if the authors intend to adapt a previously developed program (i.e GTKCP), it might be worthwhile to consider indicate such an ‘assessing the adaptability” approach in the title. This would allow the reader to gain traction on the process and protocol rather quickly. It would also be picked up by others also seeking to adapt protocols in different settings.
2. Given the that CZS is mosquito-borne, and given that not all readers might be as familiar with the pathogenesis, it may be worth a very brief description of the transmission vectors.
3. While the stages of the protocol are very clear, it remains somewhat a complex interplay in time and space. The authors may wish to consider providing a flow chart/figure of the protocol (along with timing and duration) so that the reader can quickly refer back and better appreciate the methods.
4. Given that this is a student protocol submission, further details on the type and form of compensation that was provided from Fiocruz to the participants. This could be a helpful model (or at least consideration) for others to consider during their implementation of similar programs in lower resourced settings.
5. There appeared to be sufficient details on the first three objectives, but there was very little in regards to the fourth objective “Update the manual.” Please provide details on this final objective so as to make the study protocol complete.
6. While I recognize that this is a study protocol submission, I was left wishing to be able to easily access the findings. I followed the ‘trail’ to access all of the consents forms, and excel files, and they were all well developed in my opinion. I fully support the authors focus on protection of personal information that might be identifiable. However, I would suggest that the authors reconsider this position and provide open access to some basic information on the population that was involved in phases of the study.  This high level aggregated information would provide context, and deepen the appreciation for all the work that has been inputted into this protocol. I have read that it was possible to contact Dr. Duttine (and all his information was well placed in the manuscript), but not all reader will likely do this extra step, and so I would encourage the authors to consider.

Overall, this is a well written study protocol, and I look forward to the subsequent publications on this important public and population health challenge.

I confirm that I have read this submission and believe that I have an appropriate level of expertise to confirm that it is of an acceptable scientific standard.
